# Origin of Discrete and Continuous Dark Noise in Rod Photoreceptors

**DOI:** 10.1523/ENEURO.0390-23.2023

**Published:** 2023-11-27

**Authors:** Ulisse Bocchero, Johan Pahlberg

**Affiliations:** Photoreceptor Physiology Group, National Eye Institute, National Institutes of Health, Bethesda, Maryland 20892-2510

**Keywords:** cGMP concentration, continuous noise, discrete noise, rod photoreceptor, thermal activation, visual pigment

## Abstract

The detection of a single photon by a rod photoreceptor is limited by two sources of physiological noise, called discrete and continuous noise. Discrete noise occurs as intermittent current deflections with a waveform very similar to that of the single-photon response to real light and is thought to be produced by spontaneous activation of rhodopsin. Continuous noise occurs as random and continuous fluctuations in outer-segment current and is usually attributed to some intermediate in the phototransduction cascade. To confirm the origin of these noise sources, we have recorded from retinas of mouse lines with rods having reduced levels of rhodopsin, transducin, or phosphodiesterase. We show that the rate of discrete noise is diminished in proportion to the decrease in rhodopsin concentration, and that continuous noise is independent of transducin concentration but clearly elevated when the level of phosphodiesterase is reduced. Our experiments provide new molecular evidence that discrete noise is indeed produced by rhodopsin itself, and that continuous noise is generated by spontaneous activation of phosphodiesterase resulting in random fluctuations in outer-segment current.

## Significance Statement

Retinal rod photoreceptors display quantum sensitivity, and two prominent sources of noise called discrete and continuous noise have been considered to be potentially limiting for the detection of single photons. Our study provides direct molecular evidence of the origin of dark noise in rod photoreceptors, confirming spontaneously activated rhodopsin as the origin of discrete noise and spontaneous activation of phosphodiesterase as the most likely source of the continuous noise.

## Introduction

Our sense of vision is initiated by the absorption of photons by visual pigment molecules in our retinal photoreceptor cells, the rods and the cones. While cones are less sensitive than rods, requiring hundreds of absorbed photons to elicit a response, a dark-adapted rod is capable of generating a detectable response to a single-photon absorption (Baylor et al., 1979), and must transmit these detections to the rest of the visual system, ultimately producing conscious perception (Field et al., 2005; Field and Sampath, 2017). Amplification in the transduction machinery inevitably introduces noise, as stochastic events unrelated to the signal are also amplified. This noise is passed forward through the retinal circuitry and threatens to obscure the detection of physiologically meaningful signals.

Two major forms of intrinsic noise are known to be present in the rod photocurrent, termed discrete and continuous noise (Baylor et al., 1980). Discrete noise events, with a time course and amplitude very similar to the single-photon response to real light, are believed to arise from the spontaneous thermal activation of rhodopsin (Rh). Continuous noise consists of low-amplitude fluctuations in the rod photocurrent and is thought to arise in phototransduction downstream of the visual pigment (Rieke and Baylor, 1996; Pahlberg and Sampath, 2011). Despite the importance of this noise for understanding the ultimate limits to visual detection (Hecht et al., 1942; Barlow, 1956, 1957; Aho et al., 1987, 1993; Copenhagen et al., 1987; Field et al., 2005; Pahlberg and Sampath, 2011), we still cannot say with certainty how either form of noise is produced.

To identify the sources of noise in rods, we took advantage of genetically modified mouse lines with reduced expression of proteins of the transduction cascade. We recorded from rods heterozygous for the rhodopsin gene to show that the rate of discrete noise events is diminished in proportion to the decrease in rhodopsin expression. To identify the source of the continuous noise, we recorded from rods with reduced levels of transducin (Tr) and phosphodiesterase (PDE). These experiments show that continuous noise is independent of transducin concentration but markedly altered when the level of phosphodiesterase is reduced. Our experiments give direct molecular demonstration of the origin of noise in rods and provide a clearer understanding of the limits to light detection near the visual threshold.

## Materials and Methods

### Mouse lines and preparation

This study was conducted in accordance with the recommendations of the *Guide for the Care and Use of Laboratory Animals* of the National Institutes of Health (NIH), and the Association for Research in Vision and Ophthalmology Statement for the Use of Animals in Ophthalmic and Vision Research. The animal use protocol was approved by the Animal Care and Use Committees of the NIH (approval ASP 1344-21).

While wild-type (WT) mice (C57BL/6J; The Jackson Laboratory) of either sex were used in some experiments, most experiments were performed on mice with reduced Rh (Rh heterozygous, Rh^+/−^), rod Tr (Tr heterozygous, Tr^+/−^), and cGMP PDE (PDE6AB heterozygous, *PDEAB*^+/−^; Calvert et al., 2001; Mao et al., 2013; Majumder et al., 2015), bred for more than five generations into a C57BL/6J background. The retinas of these mice do not degenerate up to 36 weeks of age and display robust rod-driven responses in bipolar cells indicating near-normal rod phototransduction and synaptic transmission. To count discrete events, the WT and *Rh*^+/−^ mice were bred into a guanylate cyclase activating proteins (*GCAPs*^−/−^) background, which allows single-photon events to be easily counted because of the elimination of Ca^2+^-dependent feedback in phototransduction (Burns et al., 2002; Okawa et al., 2010).

Mice were maintained on a 12 h light/dark cycle and were always dark adapted overnight before experimentation. All experimental manipulations were performed in darkness under infrared illumination visualized with infrared image converters (B.E. Meyers). Mice were euthanized according to guidelines approved by NIH (ASP 1344–18). Following euthanasia, eyes were enucleated, the lens and cornea were removed, and eyecups were stored in darkness at 32°C in Ames’ media buffered with sodium bicarbonate (catalog #A1420, Sigma-Aldrich), and equilibrated with 5% CO_2_/95% O_2_. Before recordings, the retinas were isolated from the retinal pigment epithelium to prevent regeneration of the visual pigment rhodopsin.

### Protein expression

To assess the expression of Rh in our WT, *Rh*^+/−^, *PDE6AB*^+/−^, Rh Knock-out (*Rh*^−/−^), and PDE6AB Knock-out (*PDE6AB*^−/−^) lines, we performed an SDS-PAGE assay. Briefly, the retinas were extracted after mice were dark adapted overnight. The tissues were lysed using 50 µl of radioimmunoprecipitation assay buffer (in mm: 50 Tris HCl, pH 7.4; 150 NaCl; 1 mm EDTA; 1 mm EGTA; 0.1% SDS; 1% NP-40; 0.5% Na deoxycholate; protease inhibitor 40 µl/ml; and phosphatase inhibitor 100 µl/ml) per retina. The retinas were mechanically dissociated, incubated on ice for 30 min, and then centrifuged at 10,000 × *g* for 10 min at 4°C. The supernatant was separated from the pellet, and the protein concentration was quantified with the BIO-RAD Protein Assay, using BSA as a standard. Twenty micrograms of protein was diluted in Laemmli sample buffer (4% SDS, 100 mm dithiothreitol, 20% glycerol, 0.004% bromophenol blue, and 125 mm Tris-HCl, pH 6.8), and was resolved on 5–12% SDS-PAGE precast gel (Thermo Fisher Scientific) and run for 1 h at room temperature. Proteins were then transferred to a polyacrylamide membrane (Roche). The membrane with the transferred protein was checked for proper transfer using red Ponceau and then incubated with BSA 2–5% in Tris-buffered saline/0.1% Tween-20 (TBST; pH 7.6) for 1 h at room temperature, to reduce nonspecific binding. The membranes were washed three times using TBST for 20 min, and then incubated with the primary antibody mouse anti-rhodopsin (catalog #MAB5316, Millipore), anti-PDE6β and PDE6γ subunit (custom made), and mouse anti-β-actin (catalog #15G5A11/E2, Sigma-Aldrich) in 1:2000 or 1:1000 dilution in TBST solution overnight at 4°C. The next day, membranes were washed three times for 10 min with TBST, and then incubated with the secondary antibody 1:10,000 for 1 h. Membranes were washed three times with TBST for 10 min and kept moist to prevent breaking. Then membranes were either developed with the chemiluminescent substrate (Thermo Fisher Scientific) and visualized using a detector (ChemiDoc, BIO-RAD; Campello et al., 2018) or developed using luminol and oxidizing solutions (Thermo Fisher Scientific), added to reveal protein expression on photographic films (Hyperfilm, GE Healthcare). The quantification of the protein expression was performed using Fiji software.

### Microspectrophotometry

The amount of photopigment in rods was assessed with a custom-built single-beam microspectrophotometer (MSP), as previously described (Nymark et al., 2012; Frederiksen et al., 2016, 2021). Briefly, a measuring beam of monochromatic light was produced by a xenon-arc light source coupled to a scanning monochromator (Cairn Research). A piece of retina was flat mounted in a perfusion chamber with the photoreceptors facing upward. The chamber was placed on a microscope stage in the beam path of the MSP. The retinal tissue was superfused with buffered Ames’ medium equilibrated with 5% CO_2_/95% O_2_ at a rate of 4 ml/min and maintained at 35–37°C. Absorption spectra were measured from a region of the retina along its edge where outer segments could be seen protruding and perpendicular to the light beam. Measurements were made over the wavelength range of 300–800 nm in 2 nm steps. The absorbance spectrum was calculated according to Beers' Law, as follows:

(1) $$\text{OD }={\text{log}}_{10}\left(\frac{I_{i}{}_{\;}}{I_{t}{}_{\;}}\right),$$

where OD is the optical density or absorbance, *I*_i_ is the light transmitted through a cell-free space adjacent to the retinal patch, and *I*_t_ is the light transmitted through the tissue. The absorbance (OD) is very nearly proportional to rhodopsin concentration. All absorbance spectra were baseline corrected, and data were analyzed with OriginPro Graphing and Analysis software (OriginLab) and IgorPro (WaveMetrics).

### Physiological recordings from rod photoreceptors

Recordings from individual cells were made with suction electrodes or by whole-cell patch clamp from dark-adapted retinal slices as described previously (Majumder et al., 2013; Pahlberg et al., 2018; Beier et al., 2022). Suction electrode recordings were made from individual rods or pieces of retina separated from the eyecup, which were placed into a recording chamber superfused with buffered Ames’ medium equilibrated with 5% CO_2_/95% O_2_ and maintained at 35–37°C. Light-evoked responses to 20 ms flashes were generated by a shutter-controlled lamp projected through a 500 nm interference filter. Discrete noise was assessed by recording dark-adapted photocurrents for 60 s, preceded and followed by a bright flash to control for overall sensitivity and stability of the recording. Discrete events were calculated from those events in complete darkness that match the average single-photon response from photocurrent measurements. Data were sampled at 100 Hz and low-pass filtered at 30 Hz.

For patch-clamp recordings from rods, the retina was embedded in low-gelling temperature agar (catalog #A-0701, Sigma-Aldrich), and retinal slices were cut on a vibrating microtome (model VT-1000S, Leica). Tissue slices were placed in a recording chamber, and superfused with bicarbonate-buffered Ames’ medium maintained at 35–37°C and equilibrated with 5% CO_2_/95%O_2_. Light-evoked responses were recorded from single rods in the voltage-clamp mode, pipette resistance was 12–14 MΩ, and holding potential was −40 mV. Light responses were obtained by delivering 20 ms flashes from a blue-green LED (λ_max_, ∼505 nm; Thorlabs). Flash strengths varied from a just measurable response to those that produced a maximal response, increasing from flash to flash by a factor of 2. Response amplitudes were then related to flash intensities, Φ, with the Hill equation, as follows:

(2) $$\frac{R}{R_{\text{max}}}=\frac{1}{1+{\left(\frac{I_{1/2}}{\Phi }\right)}^{n}},$$

where *I*_1/2_ is the value of the stimulus intensity producing a half-maximal response, and *n* is the Hill coefficient.

Continuous noise was isolated from the current fluctuations of rod photoreceptors in darkness as described previously (Baylor et al., 1980; Rieke and Baylor, 1996; Reingruber et al., 2013). Continuous noise was assessed by patch-clamp recordings of dark-adapted photocurrents for 5–10 s, followed by a bright flash to saturate the photocurrent and isolate the instrumental noise (see Fig. 4). Continuous noise was calculated by subtracting instrumental noise from dark noise recordings and performing Fourier transformation of noise traces (Sampath and Baylor, 2002). Data were sampled at 1 kHz and low-pass filtered at 300 Hz.

Average dim flash responses were calculated as the weighted average of all linear range responses (> 25% maximum amplitude). Single-photon responses were calculated as either the average dim flash response divided by the average flash strength, or from the variance-to-mean ratio of the average dim flash response, as described previously (Baylor et al., 1979). Light sensitivity was estimated as the *I*_1/2_ based on the best Hill equation fit through the intensity response data.

Unpaired, two-tailed Student’s *t* tests were performed in Excel 2021 on the physiological properties of rod photoreceptors. Data are reported as the mean ± SEM and number of cells (*N*), unless otherwise noted. All electrophysiology figures were made using Igor 8.0 from WaveMetrics.

## Results

The goal of this study was to investigate the molecular mechanisms underlying discrete and continuous noise in rod photoreceptors. Photoactivation of rhodopsin (R*) in rods results in the closure of outer-segment cyclic nucleotide-gated (CNG) channels through stimulation of cGMP hydrolysis over multiple amplification steps in the phototransduction cascade. Discrete noise consists of signals very similar in amplitude and waveform to those produced by single photons of real light (Baylor et al., 1980). Continuous noise is caused by random fluctuations in the outer-segment current in darkness and is thought to be produced by spontaneous activation of some other phototransduction protein (Rieke and Baylor, 1996). The origin of these components was investigated by direct molecular manipulation of the putative noise sources in rod photoreceptors.

### Effect of reduced pigment content on discrete noise events in rods

**Figure 1.**
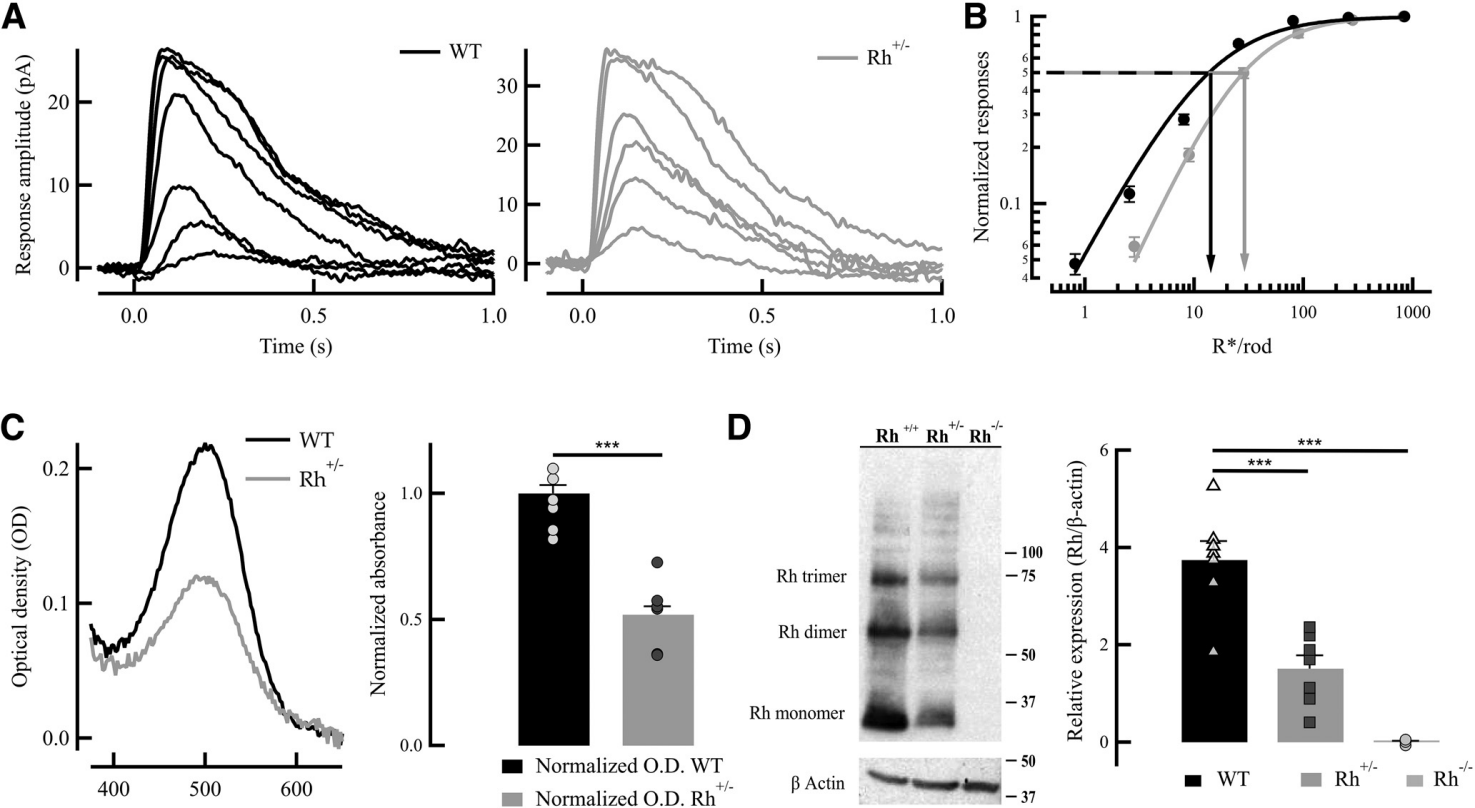
Physiological response properties of WT and *Rh*^+/−^ rods. ***A***, Representative light-evoked flash families from WT (left, black) and *Rh*^+/−^ (right, gray) rods. Patch-clamp recordings were made in whole-cell mode [membrane potential (*V*_m_) = −40 mV]. Twenty millisecond light flashes given at time 0 yielded flash strengths ranging from 1 to 800 R*/rod, increasing by a factor of 2. Recordings are representative of data collected from several experiments. ***B***, Intensity–response plot (mean ± SEM) of WT rods (black; *n* = 7)) and *Rh*^+/−^ rods (red; *n* = 7)). The sensitivity (*I*_1/2_) was derived from the best fitting Hill equation, *I*_1/2_ = 15.6 ± 0.4 and 29.8 ± 0.6, respectively. ***C***, Microspectrophotometric measurements of rhodopsin from WT rods (black) and *Rh*^+/−^ rods (red). Optical density (visual pigment content) was measured from outer segments of 12 WT rods (OD =* *0.219 ± 0.03, mean ± SEM) and 14 *Rh*^+/−^ rods (OD* *=* *0.106 ± 0.012, mean ± SEM) showing a reduction in half of pigment concentration in *Rh*^+/−^ mice (***p < 0.0001; see Materials and Methods). ***D***, Western blots for rhodopsin showing that concentration in *Rh*^+/−^ rods (1.5 ± 0.3 a.u., normalized over β-actin content.) compared with WT rods (3.7 ± 0.4 a.u., normalized over β-actin content) is reduced by half (***p < 0.001).

We first characterized the number of discrete noise events when rhodopsin expression is reduced in rhodopsin (*Rh*^+/−^) heterozygous mice. The light sensitivity of the photoreceptors in *Rh*^+/−^ mice in a common C57BL/6J background was reduced by approximately twofold (Fig. 1*A*,*B*). This result is consistent with previous measurements (Calvert et al., 2001) and would be expected from the reduction in visual pigment concentration and the consequent decrease in collecting area. The reduction in pigment concentration in our *Rh*^+/−^ mice in a C57BL6J background was confirmed by MSP (Fig. 1*C*) and Western blots (Fig. 1*D*) to be approximately half that of WT littermates (Lem et al., 1999).

**Figure 2.**
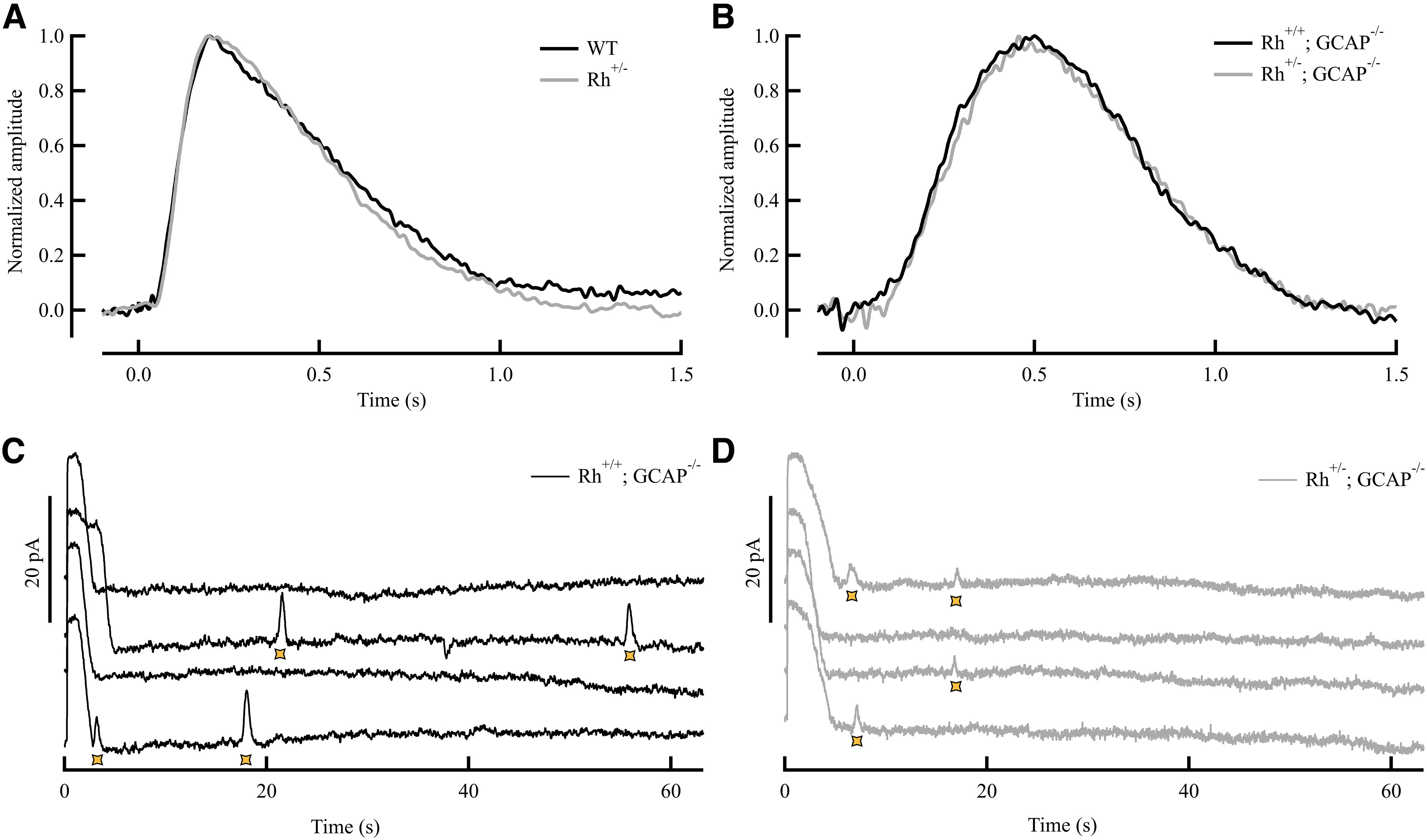
Discrete noise events in rod photoreceptors in complete darkness. ***A***, Average normalized dim-flash responses recorded with the suction electrode technique from WT (black) and *Rh* ^+/−^ (gray) rods. Responses were derived from 10 and 11 rods (>25% of maximum response amplitude), and the time-to-peak values were 246 ± 15.1 and 237.5 ± 18.8 ms, respectively (mean ± SEM). ***B***, Average normalized dim-flash responses recorded with the suction electrode technique from *R^+/+^;GCAPs*^−/−^ (black) and *Rh*^+/−^*;GCAPs*^−/−^ rods (gray), *N* = 36 and *N* = 46, respectively, and time-to-peak values were 427.9 ± 30.3 and 432.3 ± 36.8 ms, respectively. ***C***, Representative recordings of outer-segment membrane currents recorded with the suction electrode technique from dark-adapted *Rh^+/+^*;*GCAPs*^−/−^. Rods were first stimulated with a saturating light flash to demonstrate viability of the cell and then placed in darkness. Discrete noise events (*) were distinguished by “match filtering” the dark-noise recordings with the derived dim-flash response (see Materials and Methods). ***D***, Representative recordings of outer-segment membrane currents recorded with the suction electrode technique from *Rh*^+/−^;*GCAPs*^−/−^. Same as in ***C***.

*Rh*^+/−^*;GCAPs*^−/−^ mice were then used to estimate how a direct manipulation in pigment concentration would affect the discrete noise events in rods in complete darkness (see Materials and Methods). The time course of the dim flash responses in both *Rh*^+/−^ and *Rh*^+/−^*;GCAPs*^−/−^ mice showed no difference from WT and *GCAPs*^−/−^ (Fig. 2*A*,*B*), in contrast to previous studies (Calvert et al., 2001). We hypothesize that this could be a strain difference effect, where in our hands the C57BL/6J background lateral diffusion of the visual pigment does not have a significant effect on kinetics of phototransduction. These results will be addressed in more detail elsewhere (Sampath et al. unpublished data). To discriminate discrete events from background noise, recordings made in complete darkness were matched to a single-photon template, which was identical for the two types of rods (Fig. 2*C*,*D*). We first presented a bright light flash to demonstrate viability of the rod and then placed the rod in darkness. The flash saturated the light response but was too dim to have produced bleach-induced events (Lamb, 1980). After combining data from all of the cells, we counted in *Rh^+/+^;GCAPs*^−/−^ control mice 68 discrete noise events in 5052 s of recording across 10 cells, corresponding to a thermal rate of 0.013 s^−1^ rod^−1^, in good agreement with previous estimates for mouse rods with the normal complement of rhodopsin (Burns et al., 2002; Fu et al., 2008). In *Rh*^+/−^*;GCAPs*^−/−^ mice, we observed 39 events in 5965 s across 11 cells, corresponding to a thermal rate of 0.006 s^−1^ rod^−1^ (Table 1). Thus, the frequency of discrete noise events was reduced by approximately twofold in rods with half the amount of rhodopsin.

**Table 1:** Discrete noise events in rod photoreceptors

	Rods	Time	Events	Discrete rate
*Rh* ^+/+^ *;GCAPs* ^−/−^	10	5052 s	68	0.013 s^−1^
*Rh* ^+/−^ *;GCAPs* ^−/−^	11	5965 s	39	0.006 s^−1^

Collected data of discrete noise events recorded in total darkness from dark-adapted *Rh*^+/+^*;GCAPs*^−/−^; and *Rh*^+/−^*;GCAPs*^−/−^ rods.

### Effect of transducin and PDE concentrations on light response properties

**Figure 3.**
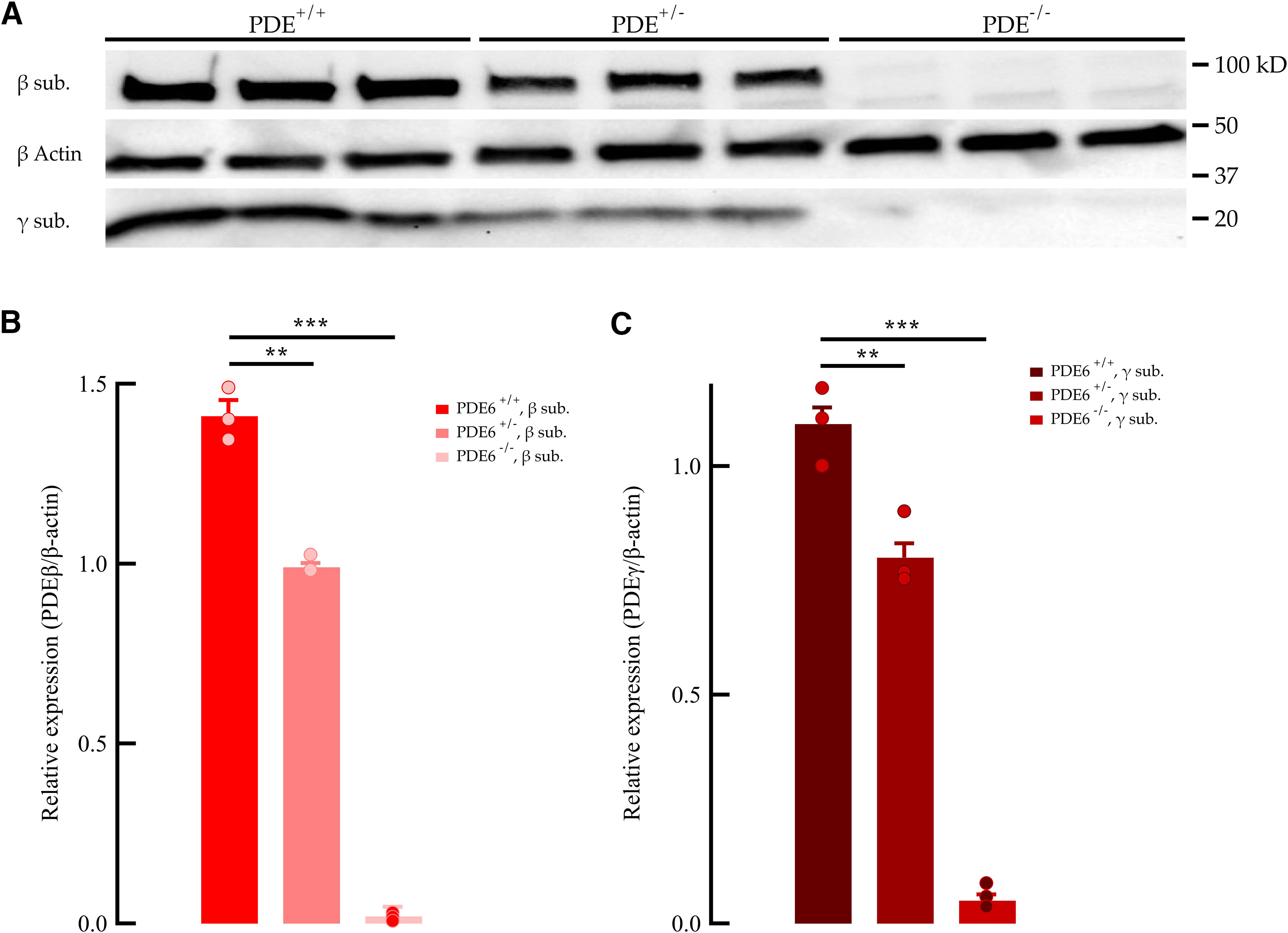
Reduced PDE6 expression in *PDEAB*^+/−^ rods. ***A***, Representative immunoblots for PDE6 β and γ subunit expression in *PDE6AB^+/+^*, *PDE6AB*^+/−^, and *PDE6AB*^−/−^ retinas. Examples of protein expression in 6 retinas (2 for each well) per genotype. ***B***, Quantification of PDE6 β subunit expression in WT (red), *PDE6AB*^+/−^ (light red), and *PDE6AB*^−/−^ (pink) rods, normalized to the β-actin loading control. The expression of the β subunit was significantly decreased in both *PDE6AB*^+/−^ rods (70% of the *PDE6AB^+/+^*, **p < 0.01, *N* = 3) and *PDE6AB*^−/−^ rods (>1% of the *PDE6AB^+/+^*, ***p < 0.001, *N* = 3). ***C***, Quantification of the PDE6 γ subunit in WT (brown), *PDEAB*^+/−^ (light brown), and *PDEAB*^−/−^ (red) rods, normalized to the β-actin loading control. The expression of the γ subunit was significantly decreased in both *PDE6AB*^+/−^ (73% compared with *PDE6AB^+/+^*, **p < 0.01, *N* = 3) and *PDE6AB*^−/−^ (>1% compared with *PDE6AB^+/+^*, ***p < 0.001, *N* = 3).

**Figure 4.**
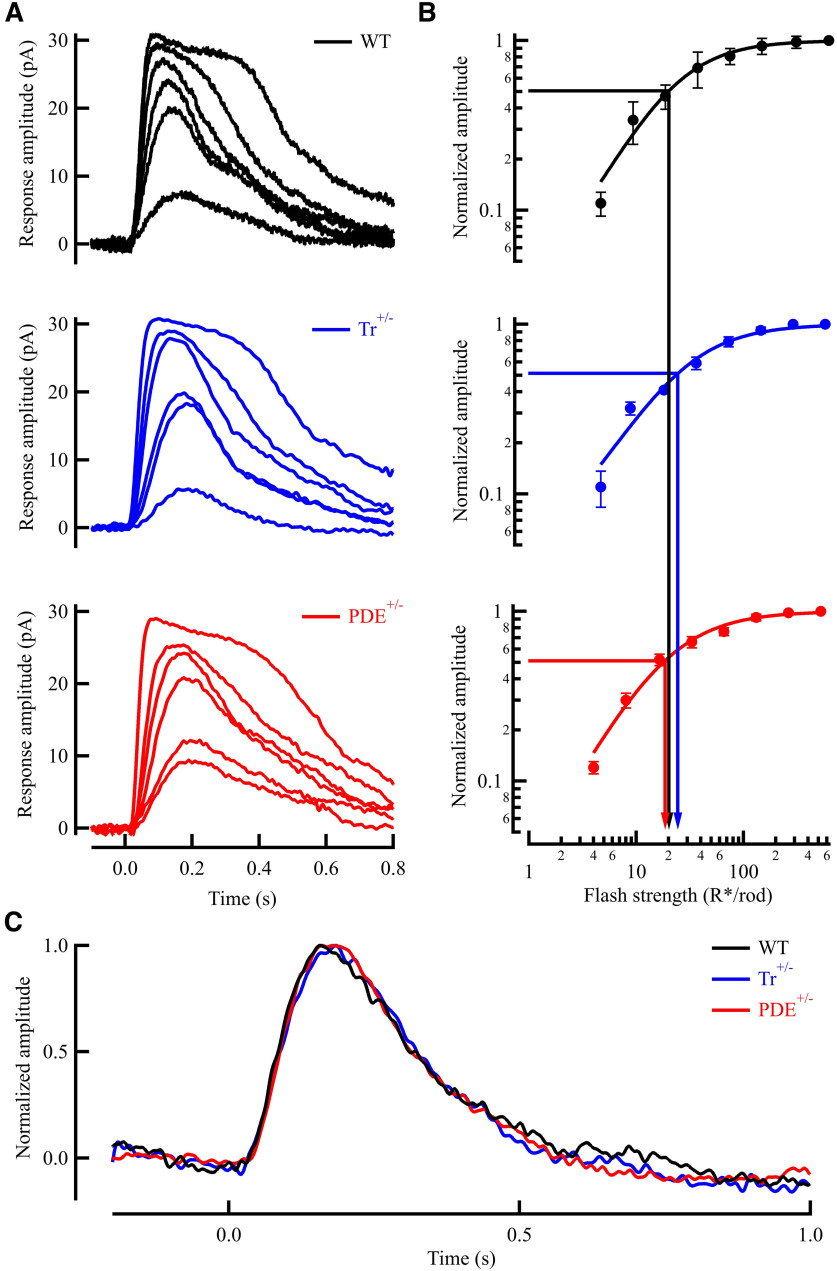
Effect of transduction protein concentration on physiological properties of rods. ***A***, Representative light-evoked responses from WT rods (C57BL/6J, black), *Tr*^+/−^ rods (blue), and *PDE6AB*^+/−^ rods (red). Patch-clamp recordings were made in whole-cell mode [membrane potential (*V*_m_) = −40 mV]. Twenty millisecond light flashes given at time 0 yielded flash strengths ranging from 4 to 550 R*/rod, increasing by a factor of 2. Reduction in expression of transducin or PDE yielded no marked change in response waveforms compared with C57BL/6J rods. Arrowheads indicate delivery time of 20 ms flashes. ***B***, Intensity–response plot (mean ± SEM) of WT rods (black, *n* = 10), *Tr*^+/−^ rods (blue, *n* = 6), and *PDE*^+/−^ (red, *n* = 11). The sensitivity (*I*_1/2_) was derived from the best fitting Hill equation, *I*_1/2_ = 19.8 ± 1.8, 23.2 ± 3.6, and 20.2 ± 3.2, respectively. Reduction in rod transducin corresponded with a small, albeit nonsignificant, decrease in flash sensitivity (*p* = 0.47). Reduction in PDE resulted in no significant change in flash sensitivity (*p* = 0.94). ***C***, Averaged single-photon responses from WT (black), *Tr*^+/−^ (blue), and *PDE*^+/−^ (red) rods, time-to-peak 222.5 ± 0.5, 245. ± 19.8, and 232. ± 32.8 ms, respectively. Reduction of neither transducin nor PDE resulted in a significant change in response kinetics.

We characterized flash response properties in wild-type mice (C57BL/6J), mice with ∼60% transducin expression (*Tr*^+/−^; Mao et al., 2013), and mice with ∼70% PDE6 expression (*PDE6*^+/−^; Fig. 3; Majumder et al., 2015) by making whole-cell voltage-clamp recordings from individual rods in retinal slices (Fig. 4*A*,*C*). The light sensitivity and time course of the dim flash response in *Tr*^+/−^ mice (Fig. 4*B*,*C*; blue) was similar compared with WT littermates (Fig. 4*B*,*C*). Again, this indicates normal rhodopsin content, as shown previously (Mao et al., 2013), and normal light-activated function of the phototransduction cascade in these rods. Next, we turned to the downstream component of the transduction cascade PDE6. Similar to our results on transducin, mice with reduced PDE concentration (Fig. 4*B*,*C*, red) showed no significant effect on light sensitivity or time course of the dim flash response when compared with WT littermates (Fig. 4*B*,*C*). Thus, neither of our specific manipulations on phototransduction molecules had any significant effect on the light response of the dark-adapted retinas to scotopic light intensities.

### Dependence of continuous noise on PDE activity and transducin expression

The single-photon response in rods must not only be separated from the discrete noise events, but also from the constantly present noise fluctuations around baseline, called continuous noise. This form of noise has been shown to arise within the rod outer segments, in the downstream transduction cascade (Baylor et al., 1980; Rieke and Baylor, 1996; Field et al., 2019). To characterize the continuous noise in rod photoreceptors, we then next examined whether a reduction in either transducin or phosphodiesterase concentration might influence the amount of dark noise. If the origin of continuous noise is controlled by either of these components of the phototransduction cascade, then a ∼1.5-fold reduction in expressed protein should affect the cGMP turnover rates through spontaneous activation.

**Figure 5.**
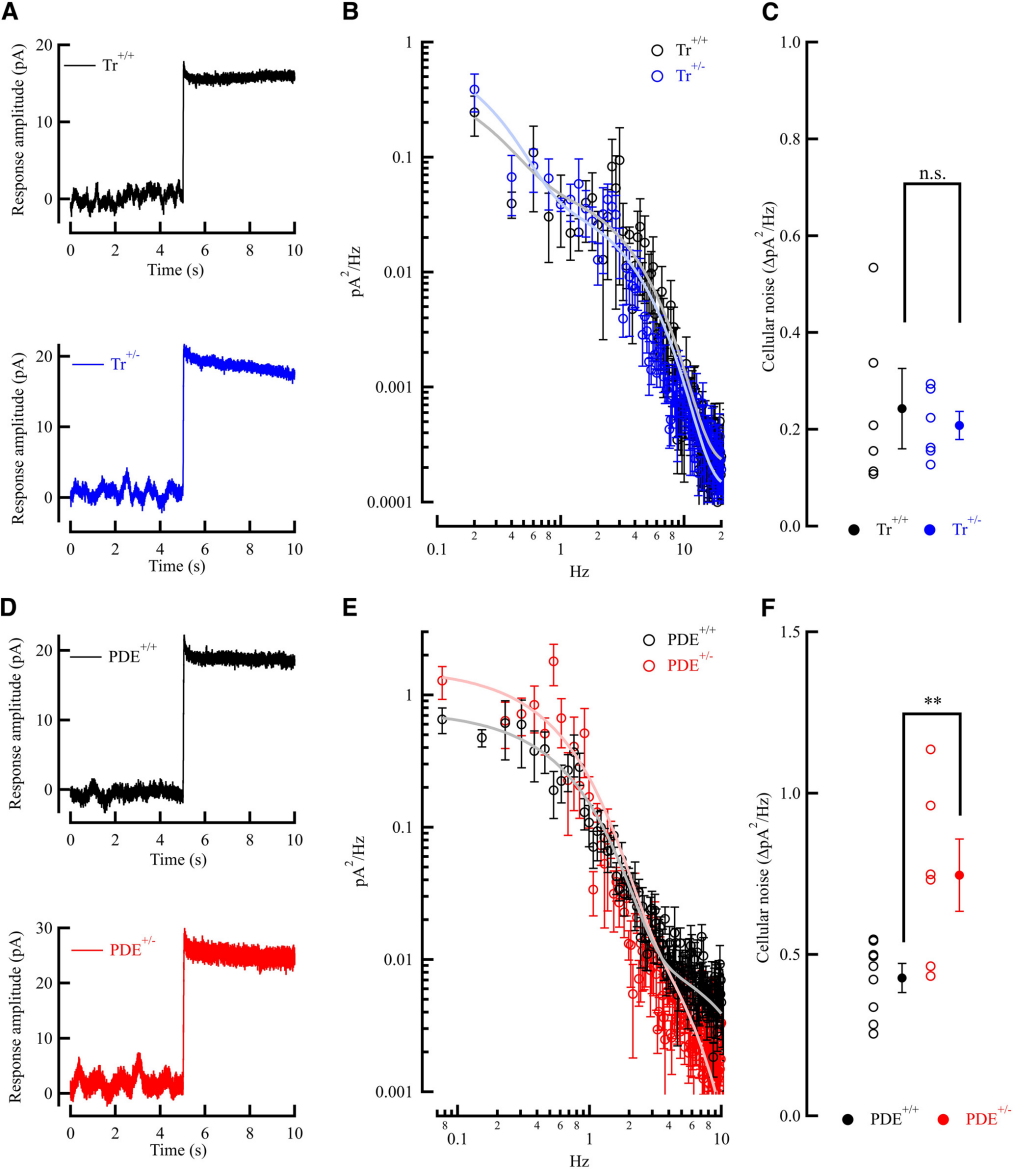
Effects of reduction in PDE and transducin expression on continuous noise. ***A***, Representative recordings of membrane currents from a WT littermate rod (black) and a *Tr*^+/−^ rod (blue). Whole-cell patch-clamp recordings were done in complete darkness, followed by a saturating light flash (6750 R*/rod) to close all of the CNG channels. ***B***, Continuous noise power spectra from WT (black, *n* = 6) and *Tr*^+/−^ (blue, *n* = 6) rods (mean ± SEM). Spectra were constructed by fast Fourier transformation of the cellular dark noise, derived by subtracting the instrumental noise (saturated response) from dark noise recordings. ***C***, Cumulative power of dark noise between 0.1 and 10 Hz. No statistical difference between WT (black) and *Tr*^+/−^ (blue) was observed. ***D***, Representative recording of membrane currents from a WT littermate (black) and PDE^+/−^ rod (red). Whole-cell patch-clamp recordings were done in complete darkness, followed by a saturating light flash (6750 R*/rod) to close all the CNG channels. ***E***, Continuous noise power spectra from WT (black, *n* = 10) and *PDE*^+/−^ (red, *n* = 10) rods (mean ± SEM). Spectra were constructed by fast Fourier transformation of the cellular dark noise, derived by subtracting the instrumental noise (saturated response) from dark noise recordings. ***F***, Cumulative power of dark noise between 0.1 and 10 Hz (mean ± SEM). A statistically significant (*p* = 0.02) approximately twofold difference in dark noise between WT (black) and PDE^+/−^ (red) was observed.

To isolate and study continuous noise, we calculated the power spectral density to generate the continuous noise spectrum for each genotype. We first investigated whether spontaneous activation of Tr can affect PDE6 activity and cGMP turnover rate, to influence the amount of continuous dark noise (Fig. 5*A–C*). To characterize the continuous noise component, patch-clamp recordings were performed without any light stimulus in complete darkness. We made whole-cell patch recordings for 5–10 s in darkness to collect all dark noise, followed by recordings in the presence of a saturating light to obtain instrumental sources of noise with transduction suppressed (Fig. 5*A*). From dark noise recordings a Fourier transform was performed, the spectrum taken in bright light was subtracted from the dark spectrum to construct power spectra of total cellular dark noise (Fig. 5*B*). The continuous-noise spectrum contained nearly all the energy within the bandwidth of 0–10 Hz, supposedly the frequency range for continuous noise (Sampath and Baylor, 2002; Field and Rieke, 2002b). Analysis of the cumulative cellular dark noise traces (Fig. 5*C*) revealed no significant differences (*p* = 0.72) in the total amount of continuous noise between *Tr*^+/−^ (Fig. 5*C*, blue) and WT littermates (Fig. 5*C*, black, Table 2), indicating that the continuous noise component in rod photoreceptors is not controlled by the spontaneous activation of Tr.

**Table 2:** Continuous noise in rod photoreceptors

	Total continuous noise	Continuous noise 0.1–1 Hz	Continuous noise 5–10 Hz
Tr^+/+^	0.24 ± 0.08 (*N* = 6)	0.1 ± 0.04 (*N* = 6)	0.0014 ± 0.007 (*N* = 5)
Tr^+/−^	0.21 ± 0.03 (*N* = 6)	0.09 ± 0.03 (*N* = 6)	0.007 ± 0.001 (*N* = 6)
PDE6^+/+^	0.42 ± 0.04 (*N* = 10)	0.33 ± 0.07 (*N* = 11)	0.029 ± 0.003 (*N* = 11)
PDE6^+/−^	0.75 ± 0.05* (*N* = 6)	0.65 ± 0.1** (*N* = 6)	0.013 ± 0.001** (*N* = 6)

The power of the cellular dark noise (pA^2^) was calculated as the frequency integral of total power in darkness subtracted by the light-isolated instrumental noise between 0.1 and 10 Hz, 0.1 and 1 Hz, and 5 and 10 Hz.

Since there was no appreciable effect of reduction of transducin, we next looked at the component downstream in the phototransduction cascade, the PDE (Fig. 5*D–F*). We again made whole-cell patch recordings for 5–10 s in darkness to collect all dark noise from PDE^+/−^ (red) and WT littermates (black), followed by recordings in the presence of a saturating light (Fig. 5*D*). In this case, power spectra analysis of the dark noise traces showed a marked redistribution of the spectral frequencies (Fig. 5*E*), with a ∼1.6-fold increase in the total amount of dark noise (*p* = 0.02) within the bandwidth of 0–10 Hz (Fig. 5*F*), consistent with our observed reduction in PDE6 concentration (Fig. 3*B*,*C*; Reingruber et al., 2013, 2015). The most prominent and significant change in frequency was an almost twofold increase between 0.1 and 1 Hz (*p* < 0.01), with an actual decrease between 5 and 10 Hz (*p* < 0.01; Table 2). Thus, a direct manipulation of PDE6 in rods had a profound effect on the spectral distribution and amount of continuous noise in these cells, whereas that of transducin did not (Table 2). These results strongly indicate the origin of continuous noise in spontaneous activation of PDE6 in mammalian photoreceptors, consistent with the transduction models previously reported (Reingruber et al., 2013) and indicate PDE activity as the dominant source of continuous noise in darkness (Rieke and Baylor, 1996).

## Discussion

The performance of the visual system near absolute threshold is limited by processes in the retina adding intrinsic noise to the light-evoked signal of absorbed photons, a concept that was formalized as the “dark light” by Barlow (1956). Baylor and colleagues (1980) first identified two sources of noise in rod photoreceptors: a discrete component of single photon-like events occurring in darkness and apparently produced by spontaneous activation of rhodopsin, and a continuous component of current noise thought to be generated by some process later in the transduction cascade. Our experiments show that *Rh*^+/−^ mice, whose rods have half the normal amount of rhodopsin, also have half the number of discrete noise events (Fig. 2), confirming spontaneously activated rhodopsin as the origin of this phenomenon. We further show that in animals with a reduction in the amount of transducin, the total power of the noise spectrum, and the frequency distribution of the power, is not significantly different from that in WT rods. In animals with a significant reduction in the amount of phosphodiesterase (Fig. 3*B*,*C*), however, total power and frequency distribution are markedly altered (Fig. 5). These experiments demonstrate that spontaneous activation of phosphodiesterase without a contribution from transducin is the most likely source of the continuous noise.

### Discrete events

We measured the frequency of discrete events in darkness and have shown that it is about half in the *Rh*^+/−^ rods as that in WT rods, in close agreement with the decrease in pigment concentration (Fig. 2*C*,*D*). This result is not unexpected, since the waveform of the discrete events in darkness is very similar to that of single-photon events produced by light, indicating that all of these signals likely have the same origin (Baylor et al., 1980; Field et al., 2005). Our experiments nevertheless provide a molecular confirmation that spontaneous activation of rhodopsin is indeed the origin of the discrete noise. The frequency of events that we have measured in WT mouse rods of 0.013 s^−1^ is in close agreement with previous estimates for mouse rods (Burns et al., 2002; Fu et al., 2008). Since there are ∼7 × 10^7^ pigment molecules per rod, this frequency is equivalent to ∼2 × 10^−10^ s^−1^ for a single rhodopsin, giving a time constant for spontaneous activation of ∼170 years.

### Continuous noise

Our experiments confirm for mammalian rods the observations of Rieke and Baylor (1996), who investigated the origin of continuous noise in amphibian rods. They showed that the continuous noise was not altered when outer-segment Ca^2+^ concentration was either buffered or prevented from changing, excluding Ca^2+^-regulated events such as synthesis of cGMP by guanylyl cyclase. They also showed that the noise was unaffected by removal of GTP from the outer segment in a truncated rod preparation, thus effectively preventing production of spontaneously activated transducin. They further showed that the noise was unaffected by addition of exogenous PDEγ. By a process of elimination, they concluded that the noise could only be produced by spontaneously activated PDE. This conclusion has also been supported by theoretical calculations of the noise by Reingruber and colleagues (2013).

We took advantage of two genetically modified mouse lines, with decreased levels of expression of Tr or PDE. In the Tr^+/−^ line used, transducin expression was found to be reduced to ∼60% compared with WT, with no significant changes in other phototransduction proteins (Mao et al., 2013; Fig. 1). Similarly, we found the expression of PDE β and γ to be reduced to 70% and 73% of WT in our PDE6AB^+/−^ rods, respectively (Fig. 3). In agreement with Rieke and Baylor (1996), we show that a change in transducin concentration in mouse rods has no significant effect on the continuous noise. A marked effect on the noise was, however, seen in PDE6^+/−^ rods having ∼70% of the normal level of PDE6 (Fig. 3). There was a change in the noise distribution (Fig. 5) such that the noise was increased at frequencies <5 Hz, with a twofold increase between 0.1 and 1 Hz, but decreased at frequencies >5 Hz (Fig. 5*E*, Table 2).

The basis for both of these effects can be attributed to the decrease in PDE expression. The increase in noise at low frequencies is a direct result of fluctuations in PDE activity, which are larger because the concentration of PDE is lower. This is because most of the time there would be no spontaneously activated PDE in each of the spaces between disks, and the cGMP concentration in the interdiscal space would slowly increase with time. A rare spontaneous PDE activation would then produce a large reduction in cGMP concentration and a pronounced fluctuation, which would generate elevated dark noise at low frequencies (Reingruber et al., 2013, 2015).

Similarly, the decrease in noise at higher frequencies could also be directly attributable to the decrease in PDE concentration, which decreases *β_D_*, the rate of turnover of cGMP in darkness. A substantial decrease in *β_D_* would produce an increase in the dark current and a slowing of the rate of decay of the photoresponse (Abtout et al., 2021), as observed by Morshedian et al. (2022) in a mouse model with only 5–10% PDE6A expression. This decrease in decay rate could effectively low-pass filter the response and decrease frequency contributions at higher frequencies (Fig. 5*E*, Table 2). In our recordings with PDE6 expression reduced to 70% we see no change in the waveform of the dim flash response (Fig. 4*C*), but a change in the frequency distribution at higher frequencies. In sum, all of our observations are most easily explained if spontaneous activation of the PDE were directly responsible for the continuous noise, in agreement with previous evidence (Rieke and Baylor, 1996; Reingruber et al., 2013).

### Signal and noise

As expected, our recordings in mice heterozygous for the rhodopsin gene showed that the light sensitivity was reduced by approximately twofold. Surprisingly, the time course of the dim flash responses in both *Rh*^+/−^ and *Rh*^+/−^*;GCAPs*^−/−^ mice showed no difference from WT and *GCAPs*^−/−^ mice (Fig. 2*A*,*B*), which is in contrast to previous studies (Lem et al., 1999; Calvert et al., 2001). This discrepancy could be the result of a difference in mouse strains, where previous studies were made with mice in a brown background, whereas our study was performed on mice bred into a C57BL/6J background for more than five generations. The results of our study would indicate that the kinetics of the light response is not set by pigment diffusion, but rather by downstream components of the transduction cascade. Our results on the *PDE*^+/−^ mice showed no change in response kinetics, whereas Morshedian et al. (2022) observed significant kinetic changes in mice with only 5–10% of PDE6. Together with our observed changes in dark noise, it is reasonable to assume that controlling the basal activity of PDE6 and regulating [cGMP] and cGMP turnover rate, is fundamental for signal properties in rods, and PDE basal activity must be controlled to set a balance between response kinetics and continuous noise levels to optimize signal and noise properties for single-photon detection.

It is usually assumed that the limit to visual perception is set by the noise of the photoreceptor (Barlow, 1957), and that discrete events produced by spontaneous activation of rhodopsin determine the absolute threshold in dim light (Barlow, 1956; Aho et al., 1987, 1993; Donner, 1992). More careful consideration would seem, however, to indicate that this view is oversimplified, and that other sources of noise including the continuous component as well as noise from random opening and closing of cyclic nucleotide-gated channels are likely also to contribute (Field et al., 2005, 2019; Field and Sampath, 2017). The noise produced by the discrete events is irreducible: no filtering or other mechanism can eliminate the effect of these random and spontaneous events from limiting visual performance. The effects of other sources of noise can, however, be decreased by filtering at various levels of processing within the retina and throughout the visual system (Field et al., 2005; Pahlberg and Sampath, 2011). Some of these filtering mechanisms have been identified (Field and Rieke, 2002a; Sampath and Rieke, 2004; Ala-Laurila et al., 2011; Hays et al., 2021), but others must surely exist and remain to be determined.

The mice we have studied have reduced levels of discrete and continuous noise. These animals and other mouse strains with altered levels of transduction proteins (Okawa et al., 2010) may serve as useful tools to help us identify the mechanisms that filter noise from signals. Recordings from these animals in retinal cells such as bipolar and ganglion cells as well as in the CNS may help us explain how we achieve the ultimate performance of visual detection.

## Synthesis

Reviewing Editor: Matthew Grubb, King’s College London Decisions are customarily a result of the Reviewing Editor and the peer reviewers coming together and discussing their recommendations until a consensus is reached. When revisions are invited, a fact-based synthesis statement explaining their decision and outlining what is needed to prepare a revision will be listed below. The following reviewer(s) agreed to reveal their identity: NONE. Note: If this manuscript was transferred from JNeurosci and a decision was made to accept the manuscript without peer review, a brief statement to this effect will instead be what is listed below.

The revised manuscript has fully addressed all of the previous Reviewers’ concerns.

One small issue: the labels at the top of Figure 3A need to be altered in the proof stage. At the moment they all show ‘PDE-/-’!
